# ﻿Morphological and phylogenetic analyses reveal two new species and a new record of *Phyllosticta* (Botryosphaeriales, Phyllostictaceae) from Hainan, China

**DOI:** 10.3897/mycokeys.91.84803

**Published:** 2022-07-04

**Authors:** Zhaoxue Zhang, Xiaoyong Liu, Xiuguo Zhang, Zhe Meng

**Affiliations:** 1 College of Life Sciences, Shandong Normal University, Jinan, 250358, China Shandong Normal University Jinan China; 2 Shandong Provincial Key Laboratory for Biology of Vegetable Diseases and Insect Pests, College of Plant Protection, Shandong Agricultural University, Taian, 271018, China Shandong Agricultural University Taian China

**Keywords:** multigene phylogeny, new species, taxonomy

## Abstract

The fungal genus *Phyllosticta* has been reported from all around the world and accommodates numerous pathogenic and endophytic species isolated from a wide range of plant hosts. Based on multilocus phylogenies from a combined dataset of genes encoding internal transcribed spacer (ITS), large subunit of ribosomal RNA (LSU rDNA), translation elongation factor 1 alpha (TEF1α), actin (ACT) and glycerol-3-phosphate dehydrogenase (GPDH), in conjunction with morphological characteristics, we describe two new species *P.oblongifoliae***sp. nov.** and *P.pterospermi***sp. nov.**, as well as a new Chinese record *P.capitalensis*. Their similarity and dissimilarity to morphologically-allied and phylogenetically-related species are also annotated and discussed.

## ﻿Introduction

*Phyllosticta* Pers. was introduced by Persoon (1818) and *P.convallariae* Pers. was designated as the type species (Donk 1968). Since *Phyllosticta* is distinct from other genera in that family, Seaver (1922) treated it in the family Phyllostictaceae Fr. of the order Phyllostictites. Nevertheless, *Phyllosticta* was accommodated in the family Botryosphaeriaceae Theiss. & Syd. (in Botryosphaeriales C.L. Schoch et al.) in several major studies (e.g. Crous et al. 2006; Schoch et al. 2006; Liu et al. 2012). However, the phylogenetic analyses by Wikee et al. (2013a) allocated *Phyllosticta* in a clade sister to Botryosphaeriaceae. As a result, the genus is currently accepted in the family Phyllostictaceae, in the order Botryosphaeriales.

A total of 3,213 names are documented for *Phyllosticta* in the Index Fungorum (accessed on 31 March 2022) (Hongsanan et al. 2020; Wijayawardene et al. 2020). However, many of these names have been synonymised (van der Aa and Vanev 2002). Currently, 1499 species are accepted in the genus (Bánki et al. 2022). The majority of the *Phyllosticta* species are known to infect a broad range of hosts and cause plant diseases, such as leaf and fruit spots (Wikee et al. 2013a; Zhou et al. 2015; Lin et al. 2017). Van der Aa (1973) revised this genus and established his own morphological criteria, i.e. aseptate pycnidia and hyaline conidia that are usually covered by a mucoid layer and bear a single apical appendage. According to these criteria, van der Aa and Vanev (2002) re-classified *Phyllosticta* and accepted 190 species. Other species were recombined into *Asteromella* Pass. & Thüm., *Diaporthe* Fuckel, *Guignardia* Viala & Ravaz, *Leptodothiorella* Höhn. and *Phoma* Sacc. A rare tropical species from the Brazilian Cerrado, *P.xylopiae-sericeae* Furlan. & Dianese, although morphologically well documented (Furlanetto and Dianese 1998), remains to be molecularly characterised. Recently, DNA sequencing of orthologous genes has greatly improved our knowledge of fungal phylogeny. Since van der Aa and Vanev (2002), several studies have shown that phylogenetic analyses can help delineate species in *Phyllosticta* (Baayen et al. 2002; Wulandari et al. 2009; Glienke et al. 2011; Wikee et al. 2011). More recently, new species of *Phyllosticta* have been increasingly described, based on a combination of molecular data and morphological features (Su and Cai 2012; Wang et al. 2012, 2013; Wong et al. 2012; Zhang et al. 2012, 2013; Wikee et al. 2013a; Wulandari et al. 2013; Crous et al. 2014, 2015, 2016, 2017, 2018, 2019, 2021; Zhou et al. 2015; Guarnaccia et al. 2017; Lin et al. 2017; Hattori et al. 2020; Norphanphoun et al. 2020). Norphanphoun et al. (2020) assembled all species denoted as *Phyllosticta* in GenBank, analysing a comprehensive dataset of five loci and consequently proposing six species complexes, viz. *P.capitalensis* species complex, *P.concentrica* species complex, *P.cruenta* species complex, *P.owaniana* species complex, *P.rhodorae* species complex and *P.vaccinii* species complex.

Hainan Province (18°10'–20°10'N, 108°37'–111°05'E) is an island in southern China, with an annual mean temperature of 22–27 °C and an annual precipitation of 1000–2600 mm. Bawangling National Forest Park is located in the southwest of Hainan, with a typical tropical rainforest climate. Fungi associated with leaf spots were collected from *Rhapisexcelsa*, *Garciniaoblongifolia* and *Pterospermumheterophyllum*. Using sequences of five gene loci, which include the internal transcribed spacer of ribosomal RNA (ITS rDNA), large subunit of ribosomal RNA (LSU rDNA), translation elongation factor 1 alpha (TEF1α), actin (ACT) and glycerol-3-phosphate dehydrogenase (GPDH). We also incorporated their morphology and then identified these fungi as three species of the *P.capitalensis* species complex, including two new species, as well as a species new to China, based on morphology and phylogenetic analyses.

## ﻿Materials and methods

### ﻿Isolation and morphological studies

Leaves of *Rhapisexcelsa*, *Garciniaoblongifolia* and *Pterospermumheterophyllum* showing necrotic spots were collected at the Bawangling National Forest Park, Hainan Province, China. Isolates were obtained using a tissue isolation method (Jiang et al. 2021). Fragments (5 × 5 mm) were taken from the margin of leaf lesions, surface-sterilised by immersing consecutively in 75% ethanol solution for 1 min, 5% sodium hypochlorite solution for 30 s and then rinsed in sterile distilled water for 1 min (Jiang et al. 2021). The sterilised fragments were dried with sterilised paper towels and placed on potato dextrose agar (PDA: 200 g potato, 20 g dextrose, 20 g agar, 1000 ml distilled water, pH 7.0) and incubated at 25 °C for 2–4 days. Subsequently, portions of agar with fungal mycelium from the periphery of the colonies were transferred into new PDA plates and photographed on the 7^th^ and 15^th^ days by a digital camera (Canon Powershot G7X). An inoculum of the purified colonies was placed on 2% malt extract agar (MEA:20 g malt extract, 20 g soy peptone, 15 g agar, 1000 ml distilled water, pH 5.6) and incubated under continuous near-UV light at room temperature to promote sporulation (Braun et al. 2018). Micromorphological characters were observed using an Olympus SZX10 stereomicroscope and Olympus BX53 microscope, all fitted with an Olympus DP80 high-definition colour digital camera to photo-document fungal structures. All fungal strains were stored in 10% sterilised glycerine at 4 °C for further studies. Structural measurements were taken using the Digimizer software (https://www.digimizer.com/), with thirty measurements taken for each character. The holotype specimens were deposited in the Herbarium of Plant Pathology, Shandong Agricultural University (HSAUP). Ex-holotype living cultures were deposited in the Shandong Agricultural University Culture Collection (SAUCC). Taxonomic information of the new taxa was submitted to MycoBank (http://www.mycobank.org).

### ﻿DNA extraction and sequencing

Genomic DNA was extracted from fungal mycelia grown on PDA, using a modified cetyltrimethylammonium bromide (CTAB) protocol as described in Guo et al. (2000). The internal transcribed spacer region (ITS) with intervening 5.8S rRNA gene, large subunit of rRNA gene (LSU), translation elongation factor 1-alpha gene (*tef1*), actin gene (ACT) and glyceraldehyde-3-phosphate dehydrogenase gene (GPDH) were amplified and sequenced by using the primer pairs ITS5/ITS4 (White et al. 1990), LROR/LR5 (White et al. 1990), EF1-728F/EF2 (O’Donnell et al. 1998; Carbone and Kohn 1999), ACT-512F/ACT-783R (Carbone and Kohn 1999) and Gpd1-LM/Gpd2-LM (Myllys et al. 2002), respectively.

PCR was performed using an Eppendorf Master Thermocycler (Hamburg, Germany). Amplification reactions were carried out in a 25 μl reaction volume, which contained 12.5 μl 2×Green Taq Mix (Vazyme, Nanjing, China), 1 μl of each forward and reverse primer (10 μM stock; Biosune, Shanghai, China), 1 μl template genomic DNA (approximately 10 ng/μl) and 9.5 μl distilled deionised water. PCR parameters were as follows: 94 °C for 5 min; 35 cycles of denaturation at 94 °C for 30 s, annealing at a suitable temperature for 50 s and extension at 72 °C for 1 min; and a final elongation step at 72 °C for 10 min. The suitable annealing temperatures for the genes were 55 °C for ITS, 51 °C for LSU, 52 °C for ACT, 48 °C for *tef1* and 52 °C for GPDH, respectively. PCR products were checked through a 1% agarose gel electrophoresis, stained with GelRed and visualised by a UV light. Sequencing was performed bi-directionally by Biosune Company Limited (Shanghai, China). Consensus sequences were obtained using MEGA v. 7.0 (Kumar et al. 2016). All sequences generated in this study were deposited in GenBank (Table 1).

**Table 1.** Species and GenBank accession numbers of DNA sequences used in this study.

Species^1^	Voucher^2^	Host/Substrate	Country	GenBank accession number				
				ITS	LSU	*tef1*	ACT	GPDH
*Phyllostictaacaciigena*	CPC 28295 *	*Acaciasuaveolens*	Australia	KY173433	KY173523	‒	KY173570	‒
*P.aloeicola*	CPC 21020 *	*Aloeferox*	South Africa	KF154280	KF206214	KF289193	KF289311	KF289124
	CPC 21021	*Aloeferox*	South Africa	KF154281	KF206213	KF289194	KF289312	KF289125
*P.ardisiicola*	NBRC 102261 *	*Ardisiacrenata*	Japan	AB454274	AB454274	‒	AB704216	‒
*P.aristolochiicola*	BRIP 53316 *	*Aristolochiaacuminata*	Australia	JX486129	‒	‒	‒	‒
*P.azevinhi*	MUCC0088	*Ilexpedunculosa*	Japan	AB454302	AB454302	‒	AB704226	‒
*P.beaumarisii*	CBS 535.87	*Muehlenbekiaadpressa*	Australia	AY042927	KF306229	KF289170	KF306232	KF289074
*P.brazillianiae*	LGMF 330 *	*Mangiferaindica*	Brazil	JF343572	KF206217	JF343593	JF343656	JF343758
	LGMF 333	*Mangiferaindica*	Brazil	JF343574	KF206216	JF343595	JF343658	JF343760
*P.camelliae*	MUCC0059	*Camelliajaponica*	Japan	AB454290	AB454290		AB704223	
*P.capitalensis*	CBS 128856 *	*Stanhopeagraveolens*	Brazil	JF261465	KF206255	JF261507	KF289289	JF343776
	CBS 226.77	*Baccaurearamiflora*	Brazil	FJ538336	KF206289	FJ538394	FJ538452	JF343718
	CBS 356.52	*Paphiopedilumcallosum*	Germany	FJ538342	KF206300	FJ538400	FJ538458	KF289087
	CBS 100175	*Ilex* sp.	Not given	FJ538320	KF206327	FJ538378	FJ538436	JF343699
	CBS 101228	*Citrus* sp.	Brazil	FJ538319	KF206325	FJ538377	FJ538435	KF289086
	CBS 114751	*Nepheliumlappaceum*	Hawaii	EU167584	EU167584	FJ538407	FJ538465	KF289088
	CBS 115047	*Vaccinium* sp.	New Zealand	FJ538323	KF206318	FJ538381	FJ538439	KF289077
	CBS 115049	*Aspidospermapolyneuron*	Brazil	FJ538324	KF206317	FJ538382	FJ538440	KF289084
	CBS 117118	*Bowdichianitida*	Brazil	FJ538339	JQ743603	FJ538397	FJ538455	KF289090
	CBS 120428	*Musaacuminata*	Indonesia	JN692544	KF206315	JN692532	JN692520	JN692509
	CBS 123373	*Sansevieria* sp.	Netherlands	FJ538341	JQ743604	FJ538399	FJ538457	JF343703
	CPC 13987	*Protearepens*	Portugal	KF206183	KF206281	KF289176	KF289263	KF289083
	CPC 16592	*Citruslimon*	Argentina	KF206187	KF206270	KF289273	KF289178	KF289092
	CPC 17468	*Cymbidium* sp.	Brazil	KF206188	KF206259	KF289189	KF289284	KF289120
	CPC 20256	*Ophiopogonjaponicus*	Thailand	KC291337	KF206247	KC342557	KC342534	KF289089
	CPC 20257	*Ficusbenjamina*	Thailand	KC291338	KF206246	KC342558	KC342535	KF289099
	LGMF219	*Citrussinensis*	Brazil	KF206202	KF206220	JF261490	KF289306	JF343737
	LGMF220	*Citrussinensis*	Brazil	KF206203	KF206219	JF261488	KF289307	JF343735
	LGMF222	*Citrussinensis*	Brazil	KF206204	KF206218	JF261492	KF289308	JF343739
	**SAUCC210144**	***Rhapisexcelsa***	**China**	**OM571175**	**OM571179**	**OM640045**	**OM640047**	**OM640049**
	**SAUCC210148**	***Rhapisexcelsa***	**China**	**OM571176**	**OM571180**	**OM640046**	**OM640048**	**OM640050**
*P.carochlae*	CGMCC 3.17317 *	*Caryotaochlandra*	China	KJ847422	‒	KJ847444	KJ847430	KJ847438
	CGMCC 3.17318	*Caryotaochlandra*	China	KJ847423	‒	KJ847445	KJ847431	KJ847439
*P.cavendishii*	BRIP 554196 *	*Musa* cv. Formosana	Taiwan	JQ743562	‒	KF009743	KF014080	‒
	BRIP 58008	*Banana*	Australia	KC988365	‒	KF009742	KF014071	‒
*P.cordylinophila*	CPC 20261 *	*Cordylinefruticosa*	Thailand	KF170287	KF206242	KF289172	KF289295	KF289076
	CPC 20277	*Cordylinefruticosa*	Thailand	KF170288	KF206228	KF289171	KF289301	KF289075
*P.eugeniae*	CBS 445.82	*Eugeniaaromatica*	Indonesia	AY042926	KF206288	KF289208	KF289246	KF289139
*P.fallopiae*	MUCC0113 *	*Fallopiajaponica*	Japan	AB454307	AB454307	‒	‒	‒
*P.harai*	MUCC0043	*Aucubajaponica*	Japan	AB454281	AB454281	‒	AB704219	‒
*P.hubeiensis*	CGMCC 3.14986 *	*Viburnumodoratissimim*	China	JX025037	‒	JX025042	JX025032	JX025027
	CGMCC 3.14987	*Viburnumodoratissimim*	China	JX025038	‒	JX025043	JX025033	JX025028
*P.ilicis-aquifolii*	CGMCC 3.14358 *	*Ilexaquifolium*	China	JN692538	‒	JN692526	JN692514	‒
	CGMCC 3.14359	*Ilexaquifolium*	China	JN692539	‒	JN692527	JN692515	‒
*P.maculata*	CPC 18347 *	*Musa* cv. Goly-goly pot-pot	Australia	JQ743570	‒	KF009700	KF014016	‒
	BRIP 46622	*Musa* cv. Goly-goly pot-pot	Australia	JQ743567	‒	KF009692	KF014013	‒
*P.mangiferae*	IMI 260.576 *	*Mangiferaindica*	India	JF261459	KF206222	JF261501	JF343641	JF343748
	CPC 20260	Arecaceae	Thailand	KF206193	KF206243	KF289187	KF289294	KF289114
*P.mangifera-indica*	MFLUCC 10–0029 *	*Mangiferaindica*	Thailand	KF170305	KF206240	KF289190	KF289296	KF289121
*P.miurae*	MUCC0065	*Linderapraecox*	Japan	AB454291	AB454291	‒	AB704224	‒
*P.musaechinensis*	GZAAS6.1247	*Musa*. sp.	China	KF955294	‒	KM816639	KM816627	KM816633
	GZAAS6.1384	*Musa*. sp.	China	KF955295	‒	KM816640	KM816628	KM816634
*P.musarum*	BRIP57803	*Musa*. sp.	Malaysia	JX997138	‒	KF009737	KF014055	‒
	BRIP58028	*Musa*. sp.	Australia	KC988377	‒	KF009738	KF014054	‒
***P.oblongifolae***	**SAUCC210055**	***Garciniaoblongifolia***	**China**	**OM248442**	**OM232085**	**OM273890**	**OM273894**	**OM273898**
	**SAUCC210054**	***Garciniaoblongifolia***	**China**	**OM248443**	**OM232086**	**OM273891**	**OM273895**	**OM273899**
	**SAUCC210053**	***Garciniaoblongifolia***	**China**	**OM248444**	**OM232087**	**OM273892**	**OM273896**	**OM273900**
	**SAUCC210052** *	***Garciniaoblongifolia***	**China**	**OM248445**	**OM232088**	**OM273893**	**OM273897**	**OM273901**
*P.paracapitalensis*	CPC 26517 *	*Citrusfloridana*	Italy	KY855622	KY855796	KY855951	KY855677	KY855735
	CPC 26518	*Citrusfloridana*	Italy	KY855623	KY855797	KY855952	KY855678	KY855736
	CPC 26700	*Citrusfloridana*	Italy	KY855624	KY855798	KY855953	KY855679	KY855737
	CPC 26701	*Citrusfloridana*	Italy	KY855625	KY855799	KY855954	KY855680	KY855738
	CPC 26805	*Citrusfloridana*	Italy	KY855626	KY855800	KY855955	KY855681	KY855739
	CPC 26806	*Citrusfloridana*	Italy	KY855627	KY855801	KY855956	KY855682	KY855740
	CPC 28120	*Citruslimon*	Spain	KY855628	KY855802	KY855957	KY855683	KY855741
*P.paracapitalensis*	CPC 28121	*Citruslimon*	Spain	KY855629	KY855803	KY855958	KY855684	KY855742
	CPC 28122	*Citruslimon*	Spain	KY855630	KY855804	KY855959	KY855685	KY855743
	CPC 28123	*Citruslimon*	Spain	KY855631	KY855805	KY855960	KY855686	KY855744
	CPC 28127	*Citruslimon*	Spain	KY855632	KY855806	KY855961	KY855687	KY855745
	CPC 28128	*Citruslimon*	Spain	KY855633	KY855807	KY855962	KY855688	KY855746
	CPC 28129	*Citruslimon*	Spain	KY855634	KY855808	KY855963	KY855689	KY855747
*P.parthenocissi*	CBS 111645 *	*Parthenocissusquinquefolia*	USA	EU683672	‒	JN692530	JN692518	‒
*P.partricuspidatae*	NBRC 9466 *	*Parthenocissustricuspidata*	Japan	KJ847424	‒	KJ847446	KJ847432	KJ847440
	NBRC 9757	*Parthenocissustricuspidata*	Japan	KJ847425	‒	KJ847447	KJ847433	KJ847441
*P.philoprina*	CBS 587.69	*Ilexaquifolium*	Spain	KF154278	KF206297	KF289206	KF289250	KF289137
	CBS 616.72	*Ilexaquifolium*	Germany	KF154279	KF206296	KF289205	KF289251	KF289136
***P.pterospermi***	**SAUCC210104** *	***Pterospermumheterophyllum***	**China**	**OM249954**	**OM249956**	**OM273902**	**OM273904**	**OM273906**
	**SAUCC210406**	***Pterospermumheterophyllum***	**China**	**OM249955**	**OM249957**	**OM273903**	**OM273905**	**OM273907**
*P.rhizophorae*	NCYUCC 19–0352 *	*Rhizophorastylosa*	Taiwan	MT360030	MT360039	‒	MT363248	MT363250
	NCYUCC 19–0358	*Rhizophorastylosa*	Taiwan	MT360031	MT360040	‒	MT363249	MT363251
*P.schimae*	CGMCC 3.14354 *	*Schimasuperba*	China	JN692534	‒	JN692522	JN692510	JN692506
*P.schimicola*	CGMCC 3.17319 *	*Schimasuperba*	China	KJ847426	‒	KJ847448	KJ847434	KJ854895
	CGMCC 3.17320	*Schimasuperba*	China	KJ847427	‒	KJ847449	KJ847435	KJ854896
*P.styracicola*	LC1642 *	*Styraxgradiflorus*	China	JX025040	‒	JX025045	JX025035	JX025030
*P.vitis-rotundifoliae*	CGMCC 3.17321	*Vitisrotundifolia*	USA	KJ847429	‒	KJ847451	KJ847437	KJ847443
	CGMCC 3.17322 *	*Vitisrotundifolia*	USA	KJ847428	‒	KJ847450	KJ847436	KJ847442

^1^Newly generated sequences in this study are in bold. ^2^Isolates marked with “*” are ex-type or ex-epitype strains.

### ﻿Phylogenetic analyses

The generated consensus sequences were subjected to BLAST searches to identify closely-related sequences in the NCBI’s GenBank nucleotide database (Zhang et al. 2000). For phylogenetic inferences, based on ITS-LSU-*tef1*-ACT-GPDH sequences, a subset of sequences from the alignments of Norphanphoun et al. (2020) was used as the backbone. Newly-generated sequences in this study were aligned with related sequences retrieved from GenBank (Table 1) using MAFFT 7 online tool with the Auto strategy (Katoh et al. 2019; http://mafft.cbrc.jp/alignment/server/). To establish the identity of the isolates at species level, phylogenetic analyses were first performed for each locus individually and then all loci were concatenated together for a unified analysis (ITS-LSU-*tef1*-ACT-GPDH).

Phylogenetic analyses were carried out with Maximum Likelihood (ML) and Bayesian Inference (BI) algorithms. The best evolutionary model for each partition was determined using MrModelTest v. 2.3 (Nylander 2004) and incorporated into the BI analyses. ML and BI run on the CIPRES Science Gateway portal (https://www.phylo.org/; Miller et al. 2012) using RAxML-HPC2 on XSEDE v. 8.2.12 (Stamatakis 2014) and MrBayes on XSEDE v. 3.2.7a (Huelsenbeck and Ronquist 2001; Ronquist and Huelsenbeck 2003; Ronquist et al. 2012), respectively. Default parameters were used for the ML analyses and the rapid bootstrapping with the automatic halt option was set for the BI analyses. Bayesian Inference included four parallel runs of 10,000,000 generations, with the stop rule option and a sampling frequency of 1,000 generations. Burn-in fraction was set to 0.25 and posterior probabilities (PP) were determined from the remaining trees. All resultant trees were plotted using FigTree v. 1.4.4 (http://tree.bio.ed.ac.uk/software/figtree) and the layout of the trees was edited in Adobe Illustrator CC 2019.

## ﻿Results

### ﻿Phylogenetic analyses

**Figure 1.**
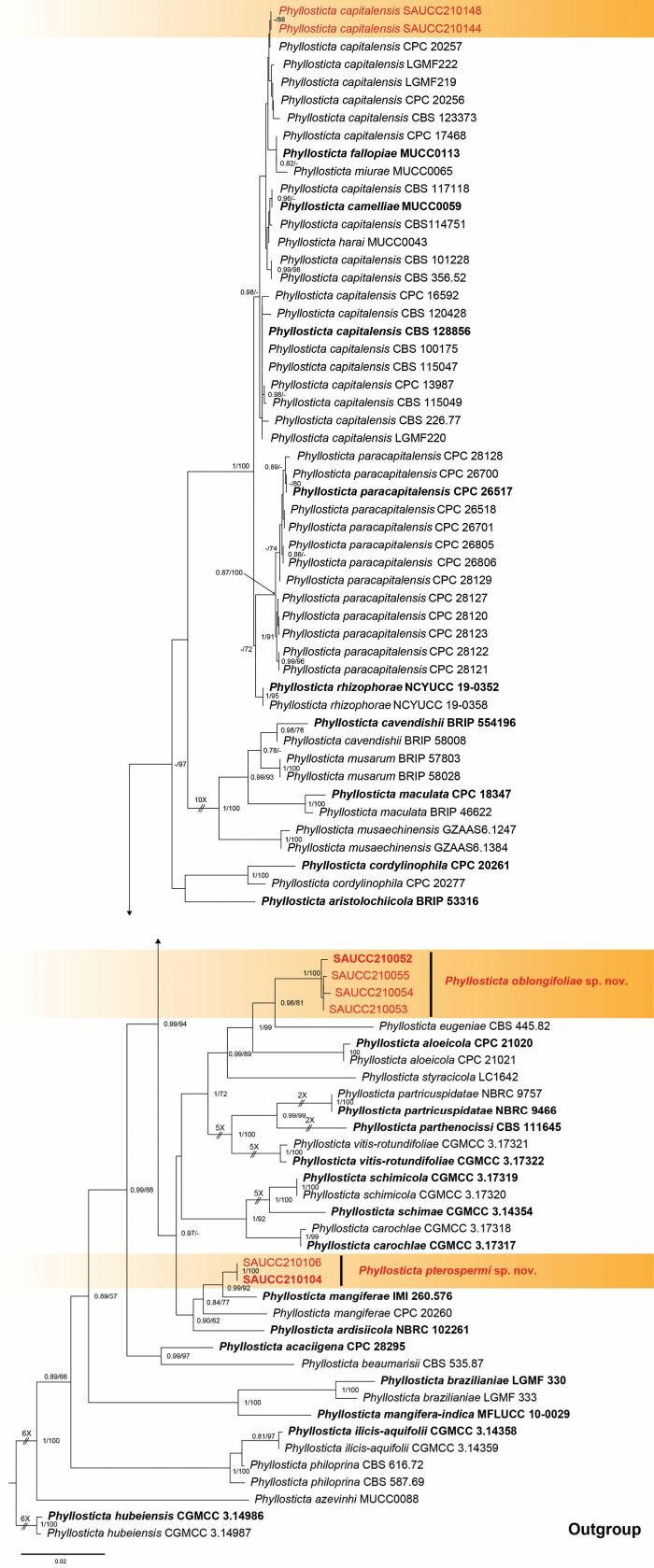
Phylogram of the *Phyllostictacapitalensis* species complex, based on a concatenated ITS, LSU, *tef1*, ACT and GPDH sequence alignment, with *Phyllostictahubeiensis* (CGMCC 3.14986, CGMCC 3.14987) of the *P.cruenta* species complex serving as outgroup. Bayesian Inference posterior probabilities and Maximum Likelihood bootstrap support values above 0.70 and 70% are shown at the first and second position, respectively. Ex-type cultures are indicated in bold face. Strains obtained in the current study are in red. Some branches are shortened for layout purposes – these are indicated by two diagonal lines with the number of times. The bar at the left-bottom represents substitutions per site.

A total of 86 isolates representing the *Phyllosticta* species were phylogenetically analysed, of which 84 isolates in the *P.capitalensis* species complex were considered as ingroup and two strains of *Phyllostictahubeiensis* (CGMCC 3.14986, CGMCC 3.14987) in the *P.cruenta* species complex were used as outgroup. The final alignment contained 2665 concatenated characters, viz. 1–733 (ITS), 734–1499 (LSU), 1500–1790 (*tef1*), 1791–2042 (ACT), 2043–2665 (GPDH). Of these characters, 1964 were constant, 126 were variable and parsimony-uninformative and 575 were parsimony-informative. MrModelTest recommended that the Bayesian Inference should use Dirichlet base frequencies for the ITS, LSU, *tef1*, ACT and GPDH data partitions. The GTR+I+G model was proposed for ITS, LSU and GPDH, while HKY+G for *tef1* and ACT. The MCMC analysis of the five concatenated genes was run for 1,520,000 generations, resulting in 30,402 trees. The initial 7,600 trees generated in the burn-in phase were discarded, while the remaining trees were used to calculate posterior probabilities in the majority rule consensus trees. The alignment contained a total of 876 unique site patterns (ITS: 358, LSU: 69, *tef1*: 170, ACT: 137, GPDH: 142). The topology of the ML tree confirmed the tree topology obtained from the Bayesian Inference and, therefore, only the ML tree is presented (Fig. 1). The 86 strains were assigned to 34 species, based on the five-gene phylogeny (Fig. 1). The present study revealed three species, viz. *Phyllostictaoblongifolae* sp. nov., *P.pterospermi* sp. nov. and *P.capitalensis*. The *P.oblongifolae* sp. nov. was a sister group to *P.eugeniae* (0.98/81) and the *P.pterospermi* sp. nov. was closely related to *P.mangiferae* (0.99/92).

### ﻿Taxonomy

The taxa described belong in family Phyllostictaceae.

#### ﻿ Phyllosticta oblongifoliae

Taxon classificationFungiBotryosphaerialesBotryosphaeriales

Z.X. Zhang, X.Y. Liu, Z. Meng & X.G. Zhang sp. nov.

CAC8EE11-40B5-55FF-9260-B396E6E58C74

843232

Fig. 2

##### Etymology.

The specific epithet “*oblongifoliae*” refers to the host plant *Garciniaoblongifolia*.

##### Type.

China, Hainan Province: Bawangling National Forest Park, on diseased leaves of *Garciniaoblongifolia*, 19 May 2021, Z.X. Zhang (holotype, HSAUP210052; ex-type SAUCC210052).

##### Description.

Leaf endogenic and associated with leaf spots. Asexual morph: Conidiomata pycnidial, mostly aggregated in clusters, black, erumpent. In MEA culture exuding colourless to opaque conidial masses within 10 days or longer. Pycnidial wall multilayered, textura angularis, brown to dark brown, up to 30 μm thick; inner walls hyaline. Conidiophores indistinct, often reduced to conidiogenous cells. Conidiogenous cells terminal, subcylindrical, ampulliform, hyaline, smooth, 9.0–14.0 × 2.5–4.5 μm. Conidia 8.0–13.0 × 6.0–8.0 μm, mean ± SD = 10.0 ± 1.3 × 7.2 ± 0.5 μm, hyaline, aseptate, thin and smooth walled, coarsely guttulate or with a single large central guttule, ovoid, ampulliform, ellipsoidal to subglobose, enclosed in a thin mucoid sheath, 1.0–2.0 μm thick and bearing a hyaline, apical mucoid appendage, 3.0–8.5 × 1.0–1.5 μm, flexible, unbranched, tapering towards an acutely rounded tip.

**Figure 2.**
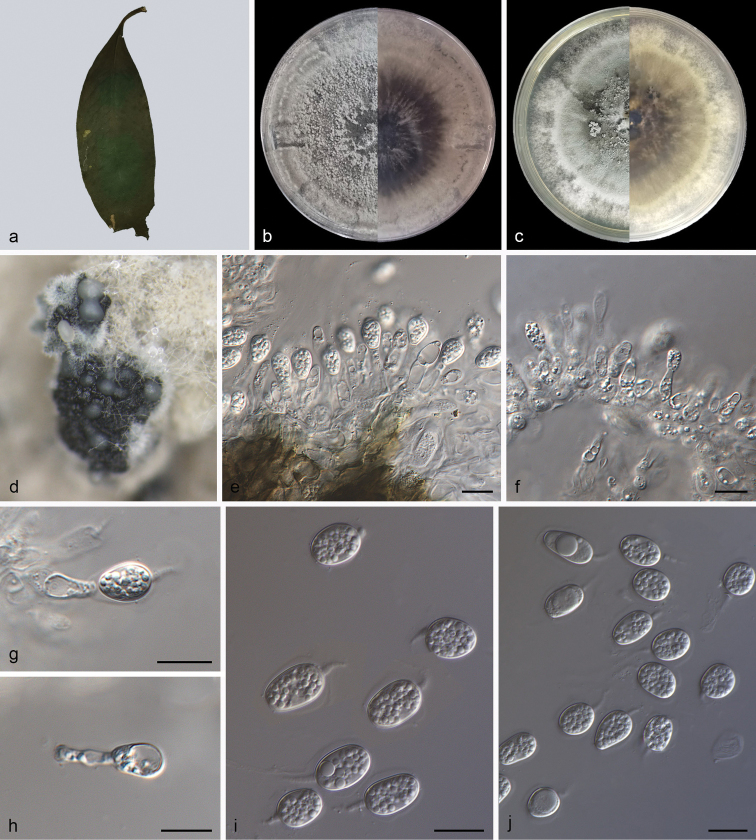
*Phyllostictaoblongifoliae* (SAUCC210052) **a** diseased leaf of *Garciniaoblongifolia***b, c** colonies (left-above, right-reverse) after 15 days on PDA (**b**) and MEA (**c**) **d** conidiomata **e–h** conidiogenous cells with conidia **i–j** conidia. Scale bars: 10 μm (**e–j**).

##### Culture characteristics

. Colonies on PDA occupying an entire 90 mm Petri dish in 14 days at 25 °C in darkness, with a growth rate of 6.0–6.5 mm/day, greenish-black in obverse and reverse. Colonies on MEA 82–86 mm in diameter after 14 days at 25 °C in darkness, with a growth rate of 5.7–6.2 mm/day, undulate at edge, white to grey white in obverse and reverse, with moderate aerial mycelia on the surface, with black, gregarious conidiomata.

##### Additional specimens examined.

China, Hainan Province: Bawangling National Forest Park, on diseased leaves of *Garciniaoblongifolia*, 19 May 2021, Z.X. Zhang, HSAUP210053, living culture SAUCC210053; on diseased leaves of *Garciniaoblongifolia*, 19 May 2021, Z.X. Zhang, paratype HSAUP210054, ex-paratype living culture SAUCC210054; on diseased leaves of *Garciniaoblongifolia*, 19 May 2021, Z.X. Zhang, paratype HSAUP210055, ex-paratype living culture SAUCC210055.

##### Notes.

*Phyllostictaoblongifoliae* is introduced, based on the multi-locus phylogenetic analysis as the strain clustered into a well-supported clade (Fig. 1; 1.00/100), which is closely related to *Phyllostictaugeniae* (0.98/81), but distinguished, based on molecular data, ITS, LSU, *tef1*, ACT and GPDH loci by 57 nucleotide differences in the concatenated alignment. Morphologically, *P.oblongifoliae* (SAUCC210052) differs from *P.ugeniae* (CBS 445.82) in its shorter and wider conidia (8.0–13.0 × 6.0–8.0 vs. 9.6–16.8 × 4.8–6.0 μm) (Wikee et al. 2013a). Therefore, we establish this fungus as a novel species (Jeewon and Hyde 2016).

#### ﻿ Phyllosticta pterospermi

Taxon classificationFungiBotryosphaerialesBotryosphaeriales

Z.X. Zhang, X.Y. Liu, Z. Meng & X.G. Zhang sp. nov.

38604F69-9BB1-52E0-B304-8DE69A1F8755

843233

Fig. 3

##### Type.

China, Hainan Province: Bawangling National Forest Park, on diseased leaves of *Pterospermumheterophyllum*, 19 May 2021, Z.X. Zhang (holotype, HSAUP210104; ex-holotype living culture SAUCC210104).

##### Etymology.

The specific epithet “*pterospermi*” refers to the genus name of the host plant *Pterospermumheterophyllum*.

##### Description.

Leaf endogenic and associated with leaf spots. Asexual morph: Conidiomata pycnidial, mostly aggregated in clusters, black, erumpent. On MEA, pycnidia exudes yellow conidial masses, within 15 days or longer. Pycnidial walls multilayered, textura angularis, brown, up to 30 μm thick; inner walls of hyaline. Conidiophores indistinct, often reduced to conidiogenous cells. Conidiogenous cells, cylindrical, hyaline, smooth, 7.5–11.0 × 2.5–4.5 μm. Conidia 8.0–12.0 × 4.5–8.5 μm, mean ± SD = 9.8 ± 0.9 × 7.3 ± 0.7 μm, hyaline, aseptate, thin and smooth-walled, coarsely guttulate or with a single large central guttule, obovoid, ellipsoidal to subglobose, enclosed in a thin mucoid sheath, 1.0–2.0 μm thick and bearing a hyaline, apical mucoid appendage, 4.0–6.8 × 1.5–3.0 μm, flexible, unbranched, tapering towards an acutely rounded tip.

**Figure 3.**
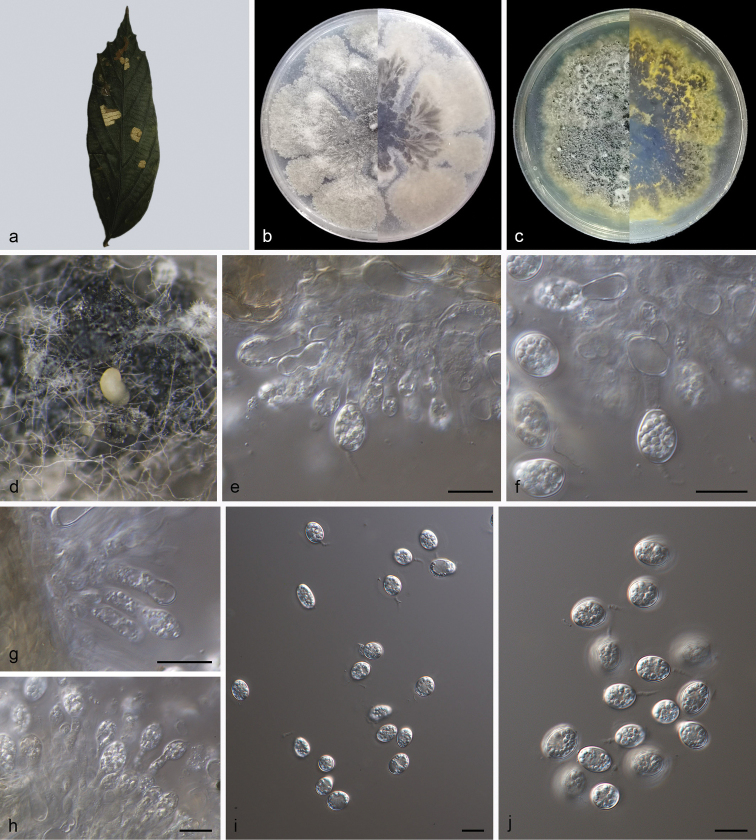
*Phyllostictapterospermi* (holotype SAUCC210104) **a** diseased leaf of *Pterospermumheterophyllum***b, c** colonies (left-above, right-reverse) after 15 days on PDA (**b**) and MEA (**c**) **d** conidiomata **e–h** conidiogenous cells with conidia **i–j** conidia. Scale bars: 10 μm (**e–j**).

##### Culture characteristics.

Colonies on PDA 80–90 mm in diameter after 14 days at 25 °C in darkness, with a growth rate of 5.7–6.5 mm/day, undulate at edge, grey white to greyish-green in obverse and reverse. Colonies on MEA 82–86 mm in diameter after 14 days at 25 °C in darkness, with a growth rate of 5.8–6.2 mm/day, undulate at edge, grey white to yellow in obverse and reverse, with moderate aerial mycelia on the surface, with black, gregarious conidiomata.

##### Additional specimen examined.

China, Hainan Province: Bawangling National Forest Park, on diseased leaves of *Pterospermumheterophyllum*. 19 May 2021, Z.X. Zhang, paratype HSAUP210106, ex-paratype living culture SAUCC210106.

##### Notes.

Two isolates from leaf spots of *Pterospermumheterophyllum* phylogenetically clustered into a well-supported clade (1.00/100), which is closely related to *P.ardisiicola* (0.90/62) and *P.mangiferae* (0.99/91; Fig. 1). However, *P.pterospermi* differs from *P.ardisiicola* by 30 nucleotides (13/603 in ITS, 3/553 in LSU and 14/248 ACT) and from *P.mangiferae* by 29 nucleotides (7/567 in ITS, 2/763 in LSU, 3/215 in *tef1*, 3/226 in ACT and 14/643 in GPDH). In morphology, they are distinguished by hosts and conidial size (8.0–12.0 × 4.5–8.5 μm in *P.pterospermi* vs. 7.0–11.0 × 5.0–7.5 μm in *P.ardisiicola* vs. 10.0–12.0 × 6.0–7.0 μm in *P.mangiferae*). Furthermore, *P.pterospermi* differs from *P.ardisiicola* and *P.mangiferae* by wider conidiogenous cells (7.5–11.0 × 2.5–4.5 μm vs. 5.0–12.5 × 1.2–2.5 μm) and from *P.mangiferae* in having longer conidiogenous cells (7.5–11.0 × 2.5–4.5 μm vs. 6.0–10.0 × 3.0–4.0 μm) (Motohashi et al. 2008; Glienke et al. 2011). Therefore, we establish this strain as *P.pterospermi* sp. nov. (Jeewon and Hyde 2016).

#### ﻿ Phyllosticta capitalensis

Taxon classificationFungiBotryosphaerialesBotryosphaeriales

Henn., Hedwigia 48: 13. 1908

1AD5AD6B-00B6-58A2-A45B-892AFC4C2BE8

Fig. 4

##### Description.

Leaf endogenic and associated with leaf spots. Asexual morph: Conidiomata pycnidial, mostly aggregated in clusters, black, erumpent. In MEA, cultures exuded colourless to opaque conidial masses, appeared on pycnidia after 10 days or longer. Pycnidial walls of multilayered, textura angularis, brown to dark brown, up to 35 μm thick; inner walls hyaline. Conidiophores subcylindrical to ampulliform, frequently reduced to conidiogenous cells or branching from a basal supporting cell, coated in mucoid layer, 8.0–14.0 × 3.0–5.0 μm. Conidiogenous cells terminal, subcylindrical to ampulliform, hyaline, smooth, 8.0–11.0 × 3.0–4.5 μm. Conidia 9.0–12.5 × 5.0–7.0 μm, mean ± SD = 10.6 ± 0.9 × 6.2 ± 0.5 μm, solitary, hyaline, aseptate, thin and smooth walled, coarsely guttulate or with a single large central guttule, ovoid, ampulliform, ellipsoidal to subglobose, enclosed in a thin mucoid sheath, 1.3–2.7 μm thick and bearing a hyaline, apical mucoid appendage, 3.0–8.5 × 1.0–1.5 μm, flexible, unbranched, tapering towards an acutely rounded tip. Spermatia hyaline, smooth, guttulate to granular, bacilliform, 6.0–8.2 × 1.3–2.0 μm, occurring in conidioma with conidia. Sexual morph: Ascomata shape and wall like those of the conidiomata. Asci bitunicate, hyaline, clavate to broadly fusoid-ellipsoid, with visible apical chamber, 2 μm diam., 45–85 × 9–13 μm. Ascospores bi- to multiseriate, hyaline, smooth, granular to guttulate, aseptate, straight, rarely curved, widest in the middle, limoniform with obtuse ends, 15–18 × 6–7 μm.

**Figure 4.**
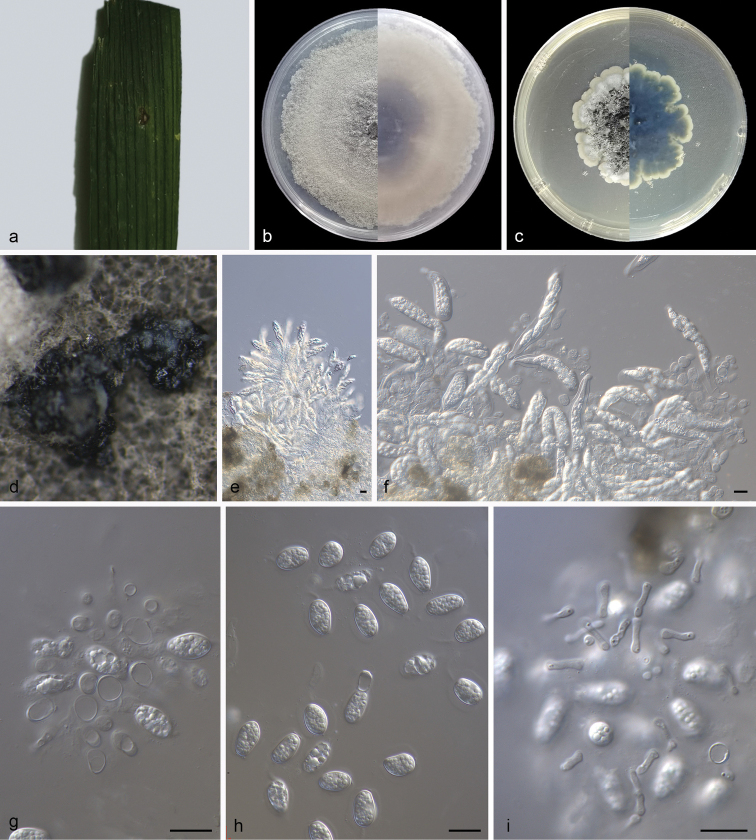
*Phyllostictacapitalensis* (holotype SAUCC210144) **a** diseased leaf of *Rhapisexcelsa***b, c** colonies (left-above, right-reverse) after 15 days on PDA (**b**) and MEA (**c**) **d** conidiomata **e** asci and ascospores **f** asci, ascospores and conidia **g** conidiogenous cells with conidia **h** conidia **i** spermatia. Scale bars: 10 μm (**e–i**).

##### Culture characteristics.

Colonies on PDA occupying an entire 90 mm Petri dish in 14 days at 25 °C in darkness, with a growth rate of 6.0–6.5 mm/day, greenish-black in obverse and reverse. Colonies on MEA 82–86 mm in diameter after 14 days at 25 °C in darkness, with a growth rate of 5.7–6.2 mm/day, undulate at edge, white to grey white in obverse and reverse, with moderate aerial mycelia on the surface, with black, gregarious conidiomata.

##### Specimens examined.

China, Hainan Province: Bawangling National Forest Park, on diseased leaves of *Rhapisexcelsa* (Thunb.) Henry ex Rehd, 19 May 2021, Z.X. Zhang, HSAUP210144, living culture SAUCC210144; on diseased leaves of *Rhapisexcelsa*. 19 May 2021, Z.X. Zhang, HSAUP210148, living culture SAUCC210148.

##### Notes.

Based on morphological features, Hennings (1908) described *Phyllostictacapitalensis* and Glienke et al. (2011) added molecular data. The holotype (CBS 128856) of *P.capitalensis* was collected from *Stanhopeagraveolens* (Glienke et al. 2011). In our current study, two isolates (SAUCC210144, SAUCC210148), collected from diseased leaves of *Rhapisexcelsa*, cluster in the *P.capitalensis* clade (Fig. 1). Although four other species are also in this clade, we consider these two isolates as *P.capitalensis*, based on their morphological characters, such as granular to guttulate ascospores (15–18 × 6–7 vs. 15–17 × 5–6 μm), subcylindrical to ampullate conidiogenous cells (8.0–11.0 × 3.0–4.5 vs. 7–10 × 3–5 μm), ellipsoidal to subglobose conidia (9–12.5 × 5–7 vs. 11–12 × 6–7 μm) and hyaline, apical mucoid appendages (3–8.5 × 1–1.5 vs. 6–8 × 1–1.5 μm).

## ﻿Discussion

Compared to other parts of China, species richness is highly diverse in Hainan Province, especially in Bawangling National Forest Park, which has a typical tropical rainforest climate. The environment favours growth of unusual microbial species. Historically, *Phyllosticta* species have been identified by morphology and host association. However, overlapping morphology makes it difficult to pinpoint homologous characters and, consequently, traditional identification of *Phyllosticta* species has long been a complicated endeavour (Norphanphoun et al. 2020). This issue has led to confusion in the taxonomy of *Phyllosticta*. Molecular phylogenetics has promoted species delimitation and species complex determination (Baayen et al. 2002; Okane et al. 2003; Motohashi et al. 2009; Wulandari et al. 2009; Glienke et al. 2011; Wikee et al. 2012). Norphanphoun et al. (2020) introduced six species complexes in *Phyllosticta*, based on five gene loci encoding the internal transcribed spacer of ribosomal RNA (ITS rDNA), large subunit of ribosomal RNA (LSU rDNA), translation elongation factor 1 alpha (TEF1α), actin (ACT) and glycerol-3-phosphate dehydrogenase (GPDH). Amongst these, the *P.capitalensis* species complex consisted of 28 cryptic species, *P.acaciigena*, *P.aloeicola*, *P.ardisiicola*, *P.aristolochiicola*, *P.azevinhi*, *P.beaumarisii*, *P.brazilianiae*, *P.capitalensis*, *P.carochlae*, *P.cavendishii*, *P.cordylinophila*, *P.eugeniae*, *P.fallopiae*, *P.ilicis-aquifolii*, *P.maculata*, *P.mangiferae*, *P.mangifera-indicae*, *P.musaechinensis*, *P.musarum*, *P.paracapitalensis*, *P.parthenocissi*, *P.partricuspidatae*, *P.philoprina*, *P.rhizophorae*, *P.schimae*, *P.schimicola*, *P.styracicola* and *P.vitis-rotundifoliae*. In this study, we focus our analyses on the *P.capitalensis* species complex and report two new species and one new Chinese record.

Multilocus phylogeny, as well as morphological characters observed in culture, described and illustrated herein eight isolates of *Phyllosticta* species from three host genera, which contributed knowledge to the diversity of *Phyllosticta* species in Hainan, China. Two new species are proposed: *P.oblongifoliae* sp. nov. and *P.pterospermi* sp. nov. This is the first time we report *Phyllosticta* species from *Pterospermumheterophyllum* (Sterculiaceae). In a recent study, *Allophomapterospermicola* was reported as pathogenic to *Pterospermum* (Marin-Felix et al. 2019). In reality, the number of phytopathogenic fungi from the *Pterospermum* host is inherently small. The known species *Phyllostictacapitalensis* (synonym *Guignardiamangiferae*; Baayen et al. 2002) was described multiple times from *Stanhopeagraveolens* (Orchidaceae) in Brazil (Glienke et al. 2011). In this study, we describe and illustrate *Phyllostictacapitalensis* again. Each of these species show typical morphological characteristics of *Phyllosticta*, i.e. conidia with mucilaginous sheaths and an apical appendage (van der Aa 1973).

*Phyllostictacapitalensis* is a cosmopolitan endophytic species reported in more than 300 host records in Fungal Databases (https://nt.ars-grin.gov/fungaldatabases/index.cfm) (Okane et al. 2001, 2003; Baayen et al. 2002; Glienke et al. 2011; Wikee et al. 2013b; Wu et al. 2014; Zhang et al. 2015; Tran et al. 2019; Hattori et al. 2020). As a weak pathogen, *P.capitalensis* causes leaf spots on tea (*Camelliasinensis*), oil palm (*Elaeisguineensis*), *Ricinuscommunis* and black spot disease on *Psidiumguajava* (Cheng et al. 2019; Nasehi et al. 2019; Liao et al. 2020; Tang et al. 2020).

## Supplementary Material

XML Treatment for Phyllosticta oblongifoliae

XML Treatment for Phyllosticta pterospermi

XML Treatment for Phyllosticta capitalensis

## Supplementary materials

Supplementary material 1 The combined ITS, LSU, tef1, ACT and GAPDH sequences Data type Phylogenetic. Explanation note The combined ITS, LSU, tef1, ACT and GAPDH sequences. https://binary.pensoft.net/file/710629This dataset is made available under the Open Database License (http://opendatacommons.org/licenses/odbl/1.0/). The Open Database License (ODbL) is a license agreement intended to allow users to freely share, modify, and use this Dataset while maintaining this same freedom for others, provided that the original source and author(s) are credited.Zhaoxue Zhang, Xiaoyong Liu, Xiuguo Zhang, Zhe Meng
